# Characterizing the Profile of Anhedonia in Individuals With Schizotypal Traits, Subthreshold Depression and Autistic Traits

**DOI:** 10.1002/pchj.827

**Published:** 2025-01-12

**Authors:** Ling‐ling Wang, Yan Gao, Chao Yan, Hui‐xin Hu, Simon S. Y. Lui, Yi Wang, Raymond C. K. Chan

**Affiliations:** ^1^ School of Psychology Shanghai Normal University Shanghai China; ^2^ Neuropsychology and Applied Cognitive Neuroscience Laboratory; CAS Key Laboratory of Mental Health, Institute of Psychology Chinese Academy of Sciences Beijing China; ^3^ Department of Psychology University of Chinese Academy of Sciences Beijing China; ^4^ Shanghai Changning Mental Health Center Shanghai China; ^5^ Key Laboratory of Brain Functional Genomics (MOE&STCSM), Shanghai Changning‐ECNU Mental Health Center, School of Psychology and Cognitive Science East China Normal University Shanghai China; ^6^ Department of Psychology, School of Humanities and Social Sciences Beijing Forestry University Beijing China; ^7^ Department of Psychiatry, School of Clinical Medicine The University of Hong Kong, Hong Kong Special Administrative Region Hong Kong China

**Keywords:** anhedonia, autistic trait, cluster analysis, schizotypal trait, subthreshold depression

## Abstract

Anhedonia is believed to be transdiagnostic symptom exist in various disorders including schizophrenia, major depressive disorder, and autism spectrum disorder. However, very few studies attempted to profile subclinical samples with schizophrenia, depressive, and autistic symptoms using measures of anhedonia scales. This study adopted a cluster analytical approach to examine the anhedonia profile in 46 individuals with schizotypal trait (ST), 43 subthreshold depression (SD), 27 autistic trait (AT), and 41 healthy controls. They completed a set of checklists capturing different dimensions of anhedonia including the anticipatory and consummatory interpersonal pleasure scale, the temporal experience of pleasure scale, the motivation and pleasure scale and the belief about pleasure scale. Cluster analysis was conducted on these measures among the merged sample of ST, SD, and AT. To validate the clusters, we administered measures on nonsocial reward processing, self‐reported empathy, and social functioning. A three‐cluster solution was found to be the best fit. Cluster 1 (*n* = 48) showed high pleasure experience, motivation, and belief about pleasure and spread evenly across three groups. Cluster 2 (*n* = 31) was characterized by low levels of anticipatory and consummatory pleasure specifically for the social domain, largely comprised of individuals with ST. Cluster 3 (*n* = 37) showed low levels of consummatory pleasure, motivation, and belief about pleasure, largely comprised of individuals with SD. The resultant clusters differed in social process and functioning. The current findings suggested distinct anhedonia subtypes within different subclinical populations. These findings may have implications for early detection and prevention for anhedonia.

## 1 Introduction

Anhedonia, that is, the diminished or loss of pleasure and motivation in formerly enjoyable activities, is a common symptom of mental disorders including schizophrenia (SCZ), major depressive disorder (MDD), and autism spectrum disorder (ASD) (American Psychiatric Association 2013; Chan et al. 2024). However, whether these disorders have shared or distinct underlying mechanism of anhedonia symptom remained unclear.

Extensive studies have adopted the transdiagnostic approach to directly compare the anhedonia among SCZ, MDD, and ASD. Overlaps of behavioral, cognitive, and neurobiological abnormalities related to anhedonia between MDD and SCZ patients have been reported (Gradin et al. 2011; Whitton, Treadway, and Pizzagalli 2015). Meanwhile, distinct anhedonia correlates have been found within SCZ, MDD, and ASD (Wang et al. 2022, 2024; Wang, Ge, et al. 2020). However, few studies have examined the anhedonia mechanisms in subclinical populations. The proposition that clinical and subclinical features may originate from the same neurobiological processes provides strong supports for studying subclinical populations (Claridge 1995). Studying subclinical populations can avoid possible confounds such as antipsychotic medications, disease chronicity, and impaired communication (Kwapil and Barrantes‐Vidal 2015). Thus, understanding the mechanism underlying anhedonia in subclinical individuals with schizotypal trait (ST), subthreshold depression (SD), and autistic trait (AT) is meaningful.

Anhedonia is a multidimensional construct which includes the anticipatory pleasure, consummatory pleasure, motivation, and belief about pleasure (Chan, Wang, and Lui 2022; Kring and Barch 2014; Strauss and Gold 2012; Treadway and Zald 2011). Moreover, anhedonia can be divided into social and nonsocial domain (Chapman, Chapman, and Raulin 1976). The presence of different components in the anhedonia construct suggests that diverse pathways can manifest as anhedonia. For example, a study has found no difference in consummatory pleasure experience between individuals with and without SD (Yang et al. 2014). By contrast, other studies have shown significantly lower consummatory pleasure experience in SD compared with controls (Liu et al. 2011; Yuan and Kring 2009). Among ST, both intact and impaired pleasure experience and motivation have been reported (Leung et al. 2010; Lui et al. 2016; McCarthy, Treadway, and Blanchard 2015; Wang et al. 2018; Wang et al. 2021; Wang, Lui, and Chan 2024). Some studies found that individuals with AT were more strongly associated with pleasure for social interactions than the relationship with general pleasure (Chevallier et al. 2012; Novacek, Gooding, and Pflum 2016). These findings suggested that AT may be associated with a selective pleasure experience deficit in the social domain. By contrast, Berthoz et al. (2013) found that AT individuals endorsed both greater physical and social anhedonia than controls (Berthoz et al. 2013). These mixed findings highlighted the need for further research to clarify the nature of anhedonia associated with ST, SD, and AT.

Among the few studies on anhedonia across ST, SD, and AT individuals, many adopted the categorical approach to directly compare the performance among different groups (Abu‐Akel et al. 2017; Zhang et al. 2021). However, the National Institute of Mental Health (NIMH) Research Domain Criteria (RDoC) project (Insel et al. 2010) preferred the dimensional approach, rather than traditional diagnostic categories, to identify transdiagnostic neural markers of anhedonia. This approach promoted the use of samples consisting of different clinical/subclinical populations combined together, to further identify subtypes that differ in profiles of biomarkers or symptomatology.

The present study aimed to characterize the anhedonia profile across different subclinical individuals with ST, SD, and AT. We hypothesized that different subtypes can be identified. Given that previous studies have found both shared and distinct mechanism of anhedonia in clinical samples of SCZ, MDD, and ASD (Gradin et al. 2011; Wang et al.  2022, 2024; Wang, Ge, et al. 2020; Whitton, Treadway, and Pizzagalli 2015), we made no prior hypothesis regarding how many clusters would be found. However, given that anhedonia has deleterious effects on functioning (Marder and Galderisi 2017), we expected that subgroups with impairments in pleasure experience, motivation, and belief about pleasure would have poorer social functioning comparing to healthy controls (HC). Moreover, we adopted tests capturing both the nonsocial reward and social processes to explore the distinct effect upon different anhedonia subtypes. We expected that different clusters would show distinct patterns of non‐social reward and social processes.

## 2 Methods

### 2.1 Participants

Forty‐six individuals with schizotypy trait (ST), 43 individuals with subthreshold depression (SD), 27 individuals with autistic trait (AT), and 41 healthy controls (HC) were recruited. ST was measured using the revised Chapman's Social Anhedonia Scale (CSAS) (Chan et al. 2012; Eckblad et al. 1982). SD was rated using the Patient health questionnaire‐9 (PHQ) (Kroenke, Spitzer, and Williams 2001; Wang et al. 2014) and the Beck Depression Inventory‐I (BDI) (Beck, Steer, and Carbin 1988; Zhang, Wang, and Qian 1990). Autistic trait was measured using the autistic questionnaire (AQ) (Baron‐Cohen et al. 2001; Zhang et al. 2016). For the inclusion criteria, the HC group has scores on all scales below the mean except for the PHQ (cut‐off score = 10) and BDI (cut‐off score = 10) of which the scores were adopted from previous suggestions (Beck, Steer, and Carbin 1988; Kroenke, Spitzer, and Williams 2001; Wang et al. 2014). The mean scores of CSAS and AQ were calculated based on a sample of 2167 college students. The cut‐off scores for these two scales were 1.5 standard deviations above the mean score. The ST group, scored 1.5 SD higher than the mean score of CSAS (score = 20), and lower than the cut‐off points of PHQ, BDI, and AQ. For the SD group, they scored above the cutoff point on both the PHQ and BDI, but lower than the cut‐off points of CSAS (score = 11) and AQ (score = 19). For the AT, they scored 1.5 SD higher than the mean score of AQ (score = 29), and lower than the cut‐off points of PHQ, BDI, and CSAS.

The recruitment was conducted in Shanghai, China. Participants first completed the online questionnaires, we then invited those participants whose satisfied the inclusion criteria of ST, SD, AT, or HC to come to our laboratory to complete the behavioral tasks. Exclusion criteria included (1) history of neurological disorders, (2) history of drug or alcohol use, (3) mental retardation or IQ < 80, and (4) personal or family history of mental disorder. The short form of the Chinese version of the Wechsler Adult Intelligence Scale (Gong 1992) was used to estimate participants' IQ. Informed consent was obtained from all participants for being included in the study. This study was approved by the Ethics Committees of the Institute of Psychology, the Chinese Academy of Sciences (H21043), the Ethics Committees of the East China normal university (HR2‐0034‐2021), and the Ethics Committees of the Shanghai Changning Mental Health Centre(M202031).

### 2.2 Measures

#### 2.2.1 Clustering Variables

All participants completed the self‐report Temporal Experience of Pleasure Scale (TEPS) (Chan et al. 2012; Gard et al. 2006) for measuring anticipatory and consummatory pleasure for general activities. Higher scores indicate higher levels of pleasure experienced. The Anticipatory and Consummatory Interpersonal Pleasure Scale (ACIPS) was used to measure both anticipatory and consummatory pleasure for interpersonal activities (Chan et al. 2010; Gard et al. 2007). Higher scores indicate higher levels of pleasure experienced. We administered the Motivation and Pleasure Scale–Self‐Report (MAP) (Llerena et al. 2013; Wang, Ma, et al. 2020) to measure participants' level of anhedonia and amotivation. Higher scores indicate higher levels of pleasure experience and motivation. The Beliefs About Pleasure Scale (BAPS) was used to assess beliefs about pleasure (Yang et al. 2018). Higher scores indicate lower levels of beliefs about pleasure.

### 2.3 Validation Variables

#### 2.3.1 Reward Process

The Effort‐Expenditure for Reward Task‐adaptive version was used to evaluate the reward process (Wang et al. 2024). It comprises two phases (decision‐making and consummatory rating). Participants were required to choose between low‐effort and high‐effort tasks. The reward magnitude of low‐effort task was￥5, while the reward magnitude of high‐effort task was selected from two ranges (narrow:￥5.4–￥6.4;wide:￥5.4–￥9.4 or narrow:￥5.4–￥6.4;wide:￥5.4–￥7.4). For every trial, a reward value was randomly selected from the range designated in the high‐effort task. After making choice, participants are asked to finish the corresponding task. Finally, they are asked to rate their pleasure experience after receiving the outcome with a nine‐point Likert scale. We calculated the total proportion of choosing high‐effort tasks, the adaptive coding to expected and outcome values (Wang et al. 2024) to be the indexes of reward process. Specifically, the adaptive coding to expected value and outcome value was calculated by taking the difference between the response slopes of the two ranges for high‐effort task during the decision‐making phase and the consummatory rating phase respectively.

#### 2.3.2 Social Process and Social Functioning

Participants completed the Chinese version of the Questionnaire of Cognitive and Affective Empathy (QCAE) (Liang et al. 2019; Reniers et al. 2011) to measure their empathy. Higher scores indicate higher empathy. The Chinese version of the Social Functioning Scale (SFS) (Lecomte et al. 2014; Wang et al. 2013) was used to measure their overall social functioning with six domains including living skills, family and friends, intimacy, interpersonal, school, and balance. Higher scores indicate better self‐reported social functioning.

## 3 Data Analysis

We implemented a hierarchical cluster analysis to the TEPS‐anticipatory, TEPS‐consummatory, ACIPS‐anticipatory, ACIPS‐consummatory, MAP total and BAPS total scores on the merged sample of three subclinical groups. The Ward's method with Z score standardization was used. Squared Euclidian distance was used as the dissimilarity measure. The dendrogram and scree plot were plotted to help visually determine the optimal cluster solution. The NbClust R package imbedded in the R statistical software version 4.0.0 was used to further verify the optimal number of clusters (Charrad et al. 2014). K‐means cluster was conducted with the optimal cluster number deduced. The group differences were examined using multivariate ANOVA. To test whether the clusters would differ in their composition of subclinical group, a chi‐square test was used.

## 4 Results

### 4.1 Demographic Information of the Sample

As shown in Table 1, the four groups did not differ significantly in their age, years of education or estimated IQ. However, they differed significantly in gender proportion (*p = 0*.03).

**TABLE 1:** The demographics and subclinical symptoms of our sample.

	ST (*n* = 46)	SD (*n* = 43)	AT (*n* = 27)	HC (*n* = 41)	*F*/*X* ^*2*^	*p*	*η* ^2^_p_
Gender (male: female)	10:36	4:39	5:22	15:26	9.41	0.02	…
Age (years)	20.07 (2.02)	19.19 (1.1)	19.41 (1.78)	19.49 (1.58)	2.28	0.08	0.04
Years of education	13.98 (1.71)	13.47 (1.01)	13.7 (1.66)	13.76 (1.63)	0.86	0.47	0.02
Estimated IQ	124.96 (8.64)	124.56 (9.02)	126.04 (9.87)	122.9 (7.5)	0.79	0.50	0.02
BDI	5.28 (2.75)	18.21 (5.74)	5.96 (2.44)	2.9 (2.68)	140.94	< 0.001	0.73
PHQ	5.11 (2.09)	14.02 (3.15)	5.52 (2.31)	3.73 (2.56)	139.39	< 0.001	0.73
AQ	22.22 (4.34)	20.58 (4.39)	30.67 (1.41)	14.15 (3.28)	107.91	< 0.001	0.68
CSAS	23.37 (2.96)	11.81 (4.86)	14.04 (3.72)	5.59 (3.12)	170.78	< 0.001	0.77

Abbreviations: AQ = autistic questionnaire; AT = autistic trait; BDI = beck depression inventory‐I; CSAS = chapman social anhedonia scale; HC = healthy control; PHQ = patient health questionnaire; SD = subthreshold depression; ST = schizotypy trait.

### 4.2 Cluster Analysis Results

According to the scree plot and the dendrogram, the optimal number of clusters was 2 or 3. The 3‐cluster solution was further verified using the Nbclust, since 8 out of the 30 clustering validity indices supported this solution.

**FIGURE 1:**
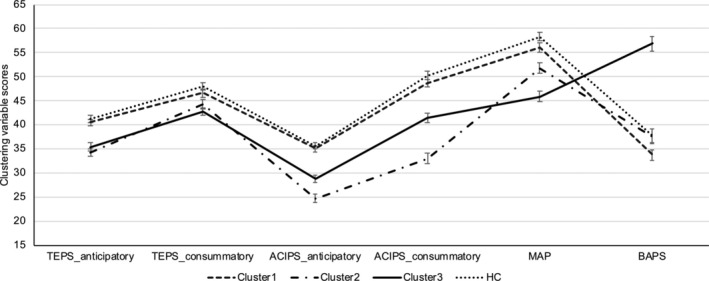
Subclinical participants' (across the three clusters) and healthy controls' performance in the clustering variables. ACIPS = anticipatory and consummatory interpersonal pleasure scale; BAPS = beliefs about pleasure scale; MAP = motivation and pleasure experience scale‐self report; TEPS = temporal experience of pleasure scale.

As shown in Table 2, Cluster 1 showed relatively higher anticipatory and consummatory pleasure experience, motivation and belief about pleasure (Figure 1). Cluster 2, however, was characterized by lower levels of anticipatory (*p* = 0.001) and consummatory pleasure (*p* < 0.001) specifically for the social domain as compared with Cluster 3. The TEPS‐consummatory score of Cluster 2 was comparable to Cluster 1 (*p* = 0.23). On the contrary, Cluster 3 showed reduced consummatory pleasure (TEPS‐consummatory) than Cluster 1 (*p* = 0.01). Moreover, Cluster 3 exhibited reduced motivation (*p* = 0.002) and belief about pleasure (*p* < 0.001) as compared to Cluster 2. Cluster 2 and Cluster 3 did not show significant difference in TEPS‐anticipatory scores. Cluster 1 exhibited comparable performances in all the clustering variables comparing to HC (*ps* < 0.05). Similarly, cluster 2 exhibited lower performance in all these variables (*ps* < 0.05) except for the belief about pleasure (*p* = 1.00) than HC.

**TABLE 2:** Subclinical participants' (in the three clusters) and healthy controls' performance in the clustering variables.

	Cluster 1 (*n* = 48)	Cluster 2 (*n* = 31)	Cluster 3 (*n* = 37)	HC (*n* = 41)	*F*(3,154)	*p*	*η* ^2^_p_	Post hoc comparison
TEPS_anticipatory	40.48 (4.64)	34.19 (4.61)	35.41 (5.7)	41.31 (4.91)	19.42	< 0.001	0.27	1 > 2; 1 > 3; 4 > 2; 4 > 3
TEPS_consummatory	46.58 (6.39)	44.23 (5.56)	42.7 (4.8)	47.95 (5.33)	6.87	< 0.001	0.12	1 > 3; 4 > 2; 4 > 3
ACIPS_anticipatory	35 (4.17)	24.65 (4.7)	28.73 (4.56)	35.64 (4.42)	51.58	< 0.001	0.50	1 > 3 > 2; 4 > 2; 4 > 3
ACIPS_consummatory	48.6 (5.69)	32.97 (6.22)	41.38 (6.2)	50.21 (5.38)	65.04	< 0.001	0.56	1 > 3 > 2; 4 > 2; 4 > 3
MAP	56.06 (7.22)	51.77 (6.11)	45.84 (6.85)	58.29 (5.93)	27.12	< 0.001	0.35	1 > 2 > 3; 4 > 2; 4 > 3
BAPS	33.77 (7.58)	37.52 (8.47)	56.84 (9.52)	37.71 (9.49)	54.86	< 0.001	0.52	3 > 1; 3 > 2; 3 > 4

Abbreviations: ACIPS = anticipatory and consummatory interpersonal pleasure scale; BAPS = beliefs about pleasure scale; HC = healthy control; MAP = motivation and pleasure experience scale‐self report; TEPS = temporal experience of pleasure scale.

We then examined whether the clusters differ in their composition of subclinical group. Results indicated significant between‐cluster differences. Specifically, Cluster 1 showed a relatively even distribution of three subclinical groups (35.4% = ST, 37.5% = SD, 27.1% = AT). Cluster 2 was mostly composed of ST individuals (64.5% = ST, 12.9% = SD, 22.6% = AT), while Cluster 3 was mostly composed of SD individuals (24.3% = ST, 56.8% = SD, 18.9% = AT).

### 4.3 Validation of the Clustering Groups

There was no significant difference in demographic variables such as education, gender, and estimated IQ among the three clusters and the HC group. However, these four groups did differ in age (*F*_(3,162)_ = 3.03, *p* = 0.03). Post hoc analysis revealed that Cluster 2 were significantly older than Cluster 1. Thus, age was taken as a covariate in the following analysis.

For reward process, no significant result was found in proportion of choosing high effort task, range adaptation to expected value and outcome value (*p*s > 0.05). For social cognition, we found a significant group difference in cognitive empathy (*F*_(3,152)_ = 5.01, *p* = 0.002). More specifically, cluster 2 were lower than HC, though it was only marginally significant (*p* = 0.05). Cluster 3 showed lower cognitive empathy as compared to both Cluster 1 (*p* = 0.04) and HC (*p* = 0.03). For the affective empathy (*F*_(3,152)_ = 2.93, *p* = 0.04), Cluster 2 and Cluster 3 showed lower scores than Cluster 1, though the between‐cluster difference was nonsignificant (*p* = 0.15; *p* = 0.13).

**FIGURE 2:**
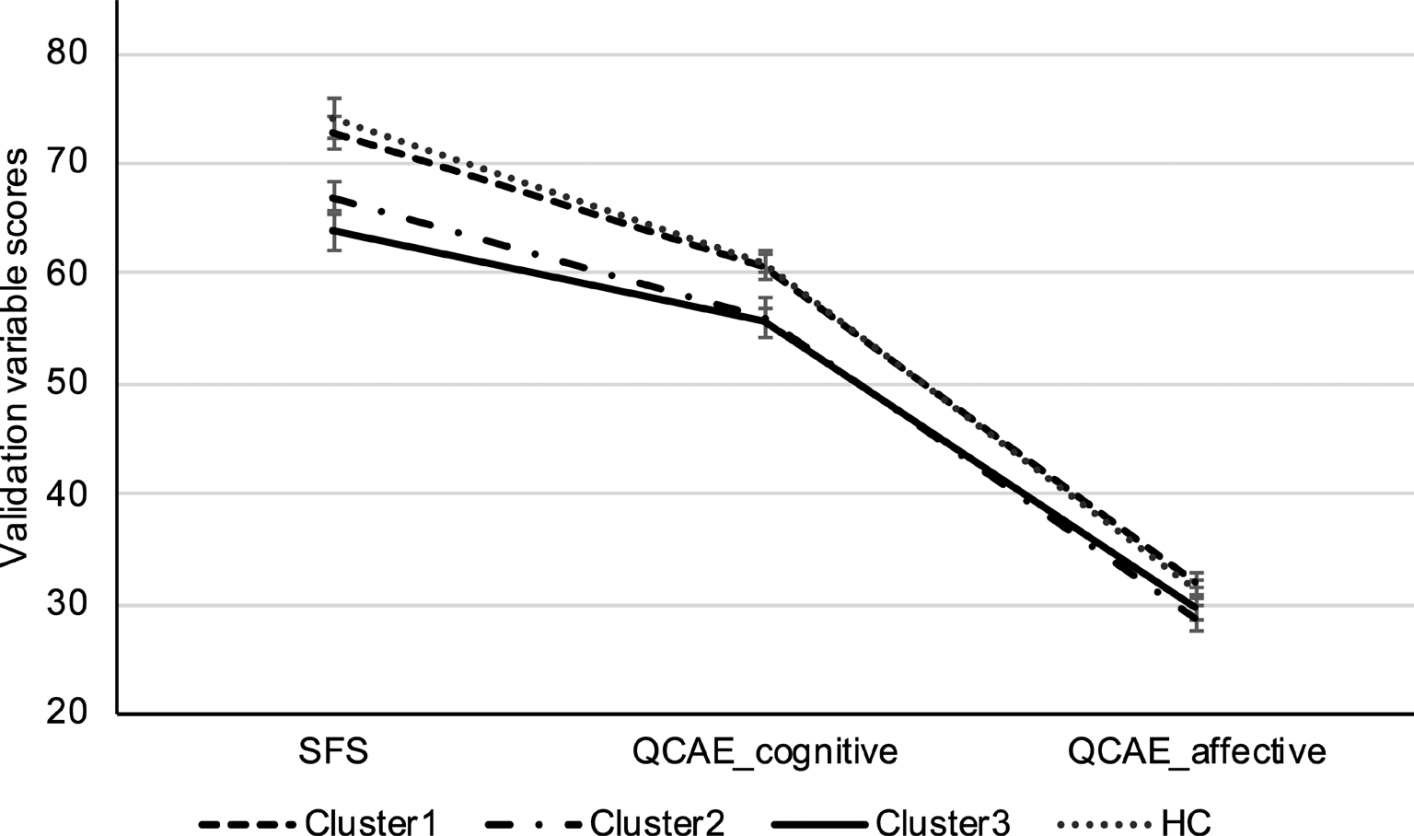
Subclinical participants' (across the three clusters) and healthy controls' performance in the validation variables. HC = healthy control; QCAE = questionnaire of cognitive and affective empathy; SFS = social functioning scale.

For the social functioning (*F*_(3,152)_ = 9.47, *p* < 0.001, Table 3), both Clusters 2 and 3 showed significantly lower scores as compared to HC (*p* = 0.03; *p* < 0.001, Figure 2).

**TABLE 3:** Subclinical participants' (in the three clusters) and healthy controls' performance in the validation variables.

	Cluster 1 (*n* = 48)	Cluster 2 (*n* = 31)	Cluster 3 (*n* = 37)	HC (*n* = 41)	*F*(3,152)	*p*	*η* ^2^_p_	Post hoc comparison
SFS	72.69 (10.12)	67.03 (7.22)	63.78 (9.64)	74.15 (10.75)	9.47	< 0.001	0.16	4 > 2; 4 > 3; 1 > 3
QCAE_cognitive	60.67 (7.85)	55.9 (10.01)	55.59 (8.48)	61.12 (7.23)	5.01	0.002	0.09	4 > 2; 4 > 3; 1 > 3
QCAE_affective	32.17 (4.71)	28.74 (6.28)	29.54 (5.12)	31.51 (4.55)	2.93	0.04	0.06	ns

Abbreviations: HC = healthy control; QCAE = questionnaire of cognitive and affective empathy; SFS = social functioning scale.

## 5 Discussion

This study examined the anhedonia subtypes using a mixed sample of subclinical individuals with ST, SD, and AT features. Cluster analysis identified three distinct groups in the mixed sample, and the clusters differed in their composition of the subclinical individuals of ST, SD, and AT. Specifically, ST and SD individuals were mainly represented in Cluster 2, which had low levels of anticipatory and consummatory pleasure in the social domain; and Cluster 3, which had low levels of consummatory pleasure, motivation, and belief about pleasure respectively. On the other hand, among 27 AT individuals, 13 of them were categorized into Cluster 1, which had relatively intact pleasure experience and motivation. Having said that, a small proportion of ST and SD individuals could be found in Cluster 1. Regarding the validation results, Cluster 2 and Cluster 3 both exhibited limited social functioning and empathy.

The current findings partially supported distinct profiles of anhedonia manifestation existing in ST, SD, and AT individuals. Yet, some individuals with ST, SD, and AT could still have intact pleasure experience, motivation, and belief about pleasure. Taken together, these findings and other previous results (Berthoz et al. 2013; Chevallier et al. 2012; Kerns, Docherty, and Martin 2008; Leung et al. 2010; Liu et al. 2011; Lui et al. 2016; McCarthy, Treadway, and Blanchard 2015; Novacek, Gooding, and Pflum 2016; Wang et al. 2018; Wang et al. 2021; Yang et al. 2014; Yuan and Kring 2009) supported a considerable heterogeneity within these disorders, as well as across ST, SD, and AT. Using an empirical approach, we provided evidence that the dimensional and categorical approach should be combined to further understanding the underlying mechanism of anhedonia. We found that Cluster 2 and Cluster 3 both showed poorer cognitive and affective empathy as compared to Cluster 1 and HC, which had relatively intact pleasure experience. The results further supported the association between anhedonia and empathy (Guo et al. 2023; Wang, Shi, et al. 2020).

As compared with Cluster 3, Cluster 2 was characterized by low levels of anticipatory and consummatory pleasure in the social domain, and mainly comprised of ST individuals. Previous meta‐analytic studies and reviews proposed that, as compared to consummatory pleasure experience, the schizophrenia spectrum is associated with more pronounced deficits in anticipatory pleasure experience (Barch, Pagliaccio, and Luking 2015; Yan et al. 2019). However, reduced consummatory pleasure experience were also reported in ST individuals, a phenomenon termed the “SCZ spectrum anhedonia paradox” (Strauss and Cohen 2018). The paradox pointed out that ST individuals have impaired consummatory pleasure experience, contrary to the intact hedonic capacity found in schizophrenia patients (Strauss and Cohen 2018). Our results were consistent with these prior findings (Strauss and Cohen 2018). However, it should be noted that previous studies defined ST without accounting for other comorbidities such as depressive symptoms and autistic traits. The current study adopted a more stringent criteria as to how we could define ST, SD, and AT individuals. Using a more carefully‐defined sample, we partially supported that ST individuals showed altered consummatory and anticipatory experience. Furthermore, this subgroup showed lower performances in social processing but not reward processing as can be seen by lower social functioning, cognitive and affective empathy scores. Such results was not surprising, because pleasure experience is associated with social processing (Buck and Lysaker 2013; Pillny, Schlier, and Lincoln 2020). Having said that, half of the ST individuals that were categorized to Cluster 2. Thus, altered consummatory and anticipatory experience may not be specific to the schizophrenia spectrum.

Cluster 3 showed reduced anticipatory pleasure, consummatory pleasure, motivation, and belief about pleasure as compared with HC. Yet, as compared with Cluster 2, Cluster 3 was characterized by low consummatory pleasure, motivation and belief about pleasure, and mainly comprised of SD individuals. Previous findings indicated that impaired consummatory and anticipatory experience is associated with depression (Barch, Pagliaccio, and Luking 2015). Diminished motivation and aberrant beliefs about pleasure are also associated with depression (Hu et al. 2018; Strunk, Lopez, and DeRubeis 2006; Treadway et al. 2012). In fact, one prior study has found that subgroups with relatively low levels in beliefs about pleasure reported more severe depressive symptoms (Wen et al. 2023). Low‐pleasure beliefs have been reported in ST individuals (Yang et al. 2018). However, after clustering analysis, we found that low‐pleasure beliefs may be a distinct anhedonia features in SD individuals. It is possible that, depressive pessimism may lead SD individuals to avoid certain challenging situations that would be rewarding, and thus result in even more low‐pleasure beliefs (Pyszczynski, Holt, and Greenberg 1987; Robinson and Clore 2002). Additionally, we found that Cluster 3 showed the lowest consummatory experience in TEPS among all groups and relatively higher anticipatory and consummatory experience during interpersonal interaction than Cluster 2. It is likely that some subclinical individuals with SD have more generalized impairments of pleasure experience, affecting the anticipatory and consummatory aspects, as well as the social and nonsocial domains. Indeed, Cluster 3 also exhibited impairments in self‐reported empathy and social functioning. However, Cluster 3 did not exhibit impairment in nonsocial reward processing. It is noteworthy that the nonsocial reward process, social process, and social functioning were measured by different levels of tasks, that is, an objective test for nonsocial reward processing but self‐report checklists for social processing and functioning. The observed differences might be related to the difference between objective and subjective measures.

Cluster 1 appeared to be a relatively “intact” subgroup. A larger proportion of AT individuals were categorized into Cluster 1. A recent review suggested that, although anhedonia is considered to be a transdiagnostic symptom, the anhedonia symptom in individuals with autistic trait may show distinct underlying mechanism (Barkus and Badcock 2019). Social anhedonia may be a consequence of impaired basic social processes such as theory of mind in people with ASD (Krach et al. 2010). In addition, a small proportion of ST and SD individuals were categorized into Cluster 1, which apparently did not exhibit anhedonia as compared to HC. It is possible that the self‐reported questionnaires we used have not captured the subtle difference between these subclinical individuals with HC. Studies using more nuanced measures such as interview or neuroimaging measures may better profile the sample.

This study has several limitations. First, our cross‐sectional design limited the conclusion we can made. Longitudinal study is required to examine the stability of these clusters and their outcomes. Besides, we only utilized the self‐reported questionnaire as clustering variables. Self‐report questionnaires may be prone to the problem of reliance on one's access to emotion knowledge (Strauss and Gold 2012). Moreover, the small number of AT participants may bias the proportion of group composition. Future study is encouraged to recruit a balanced group of participants.

Notwithstanding these limitations, this study represented an attempt to combine the categorical with the dimensional approach. Our findings partially supported the distinct anhedonia mechanism in ST, SD, and AT individuals. Besides, our findings supported a considerable heterogeneity in anhedonia symptoms, both within and across ST, SD, and AT groups.

## Conflicts of Interest

The authors declare no conflicts of interest.
